# Advances in the Oral Delivery of Protein and Peptide Drugs

**DOI:** 10.3390/pharmaceutics17050616

**Published:** 2025-05-06

**Authors:** Guanyu Chen

**Affiliations:** School of Pharmaceutical Sciences (Shenzhen), Shenzhen Campus, Sun Yat-sen University, Shenzhen 518107, China; chengy239@mail.sysu.edu.cn; Tel.: +86-020-84723750

Protein and peptide drugs (PPDs) are highly effective therapies for a wide range of diseases, including cancer, diabetes, and autoimmune disorders [1]. However, delivering these drugs orally presents significant challenges. PPDs face substantial barriers to attaining oral bioavailability, such as the rapid cleavage of PPDs by proteases in the gastrointestinal tract (GIT), and the low intestinal permeability of PPDs due to their hydrophilicity properties and high-molecular-weight structures [2]. Additionally, the denaturation of PPDs in acidic gastric environments due to their sensitivity to pH, as well as their short half-life with rapid systemic clearance, limits their therapeutic efficacy [3].

To overcome these barriers, advanced strategies for the delivery of innovative formulations are presented, such as nanocarriers. These include lipid-based carriers such as liposomes and polymeric nanoparticles; mucoadhesive systems including inorganic carriers, such as mesoporous silica, for enzymatic protection and controlled release [4]; enteric coatings such as chitosan or thiolated polymers to prolong intestinal residence [5]; absorption/permeation enhancement in the form of pH-responsive polymers (Eudragit^®^) for site-specific delivery to the small intestine [6]; targeted transport, such as SNAC (used in oral semaglutide), medium-chain fatty acids, or zonula occludens toxin, to transiently open tight junctions; chemical modification through receptor-mediated uptake (e.g., FcRn, vitamin B12 conjugation) [7]; enzyme inhibitors for PEGylation, cyclization, or D-amino acid substitution to resist proteolysis; the co-administration of protease inhibitors (e.g., aprotinin, soybean trypsin inhibitor) [8]; etc.

In the clinical translation of protein and peptide formulations, three major aspects should be considered: First, scalability, such as the manufacturing reproducibility of complex formulations (e.g., nanoparticle size uniformity). Secondly, safety, such as the long-term effects of permeation enhancers on intestinal integrity. Lastly, bioavailability, as even with advanced systems, achieving > 1% bioavailability remains a hurdle [9]. Therefore, more investigations and advanced formulation approaches are required for the oral delivery of protein and peptide drugs.

In this Special Issue on Advances in Oral Administration, twelve articles have been published, including eight research articles and four review articles. In the following sections, the highlights of these 12 articles will be summarized.

Csilla et al. conducted a study that successfully scaled up a wet grinding method using a planetary ball mill and ZrO₂ pearls, producing submicron meloxicam particles (~580 nm) with preserved crystallinity (70%) and enhanced dissolution (~100% in 5 min) without toxicity. The tenfold scale-up proved feasible by optimizing the milling parameters and excipient amounts, ensuring consistent drug quality for oral delivery (contribution 1).

Klervi et al. established a pharmacometric model which showed that CHILD-IVITAB, a child-friendly ivermectin formulation, exhibited faster and more consistent absorption than STROMECTOL^®^, enabling effective dosing for children under 15 kg, and their simulations demonstrated that a 250 µg/kg dose of CHILD-IVITAB provided an equivalent drug exposure in children < 15 kg to 200 µg/kg of STROMECTOL^®^ in adults and heavier children, supporting its use in pediatric studies (contribution 2).

Ivan et al. developed Archaeosomes, which were made from archaeal lipids via microfluidics, exhibiting exceptional stability (100 nm size, low polydispersity) and retaining > 95% of encapsulated drugs (calcein/insulin) in harsh gastrointestinal conditions, with a 35% insulin encapsulation efficiency. Their resilience during freeze-/spray-drying and their strong adhesion to intestinal cells in co-culture models highlighted their potential as stable oral drug carriers, enabling solid dosage forms for biologics like insulin (contribution 3).

Guanyu et al. developed gemcitabine-loaded β-glucan nanoparticles using film-casting and freezer-milling, achieving sustained release, enhanced intestinal permeation, and greater oral bioavailability compared to a plain drug solution. Additionally, the nanomedicine showed excellent safety characteristics and potent tumor growth inhibition in 4T1 breast cancer models, demonstrating its potential as an effective chemotherapy platform for oral gemcitabine delivery (contribution 4).

Mengyang Liu et al. developed PEGylated niosomes which significantly improved thymopentin stability and cellular uptake via active endocytosis, though the transport rates remained similar to those of the free drug, with the mechanisms shifting to energy-dependent pathways (adsorptive/clathrin-mediated) instead of passive diffusion/MRP5 efflux. The formulation, enhanced further by permeation aids (EDTA/sodium taurocholate), demonstrated promise for oral peptide delivery by balancing uptake and transport while maintaining safety, offering a potential solution to thymopentin’s metabolic instability (contribution 5).

Solene Masloh et al. introduced a novel oral delivery strategy for biotherapeutics using Nanofitin 1-F08, a robust affinity protein that targets the leptin receptor (LepR) without competing with leptin, enabling receptor-mediated transport across the intestinal barriers. Their approach demonstrated successful ex vivo transport in a porcine model, offering a promising pathway for the oral delivery of biologically active molecules while maintaining their efficacy, with the potential for future in vivo applications (contribution 6).

Else Holmfred et al. explored the compaction behavior of protein–excipient blends, revealing that lysozyme enhances the tablet strength in brittle excipients due to the improved particle bonding, while larger BSA particles reduce tensile strength, demonstrating non-linear effects with an increasing protein content. Additionally, the findings emphasized that the particulate properties of protein powders critically influence their compaction performance, showing that protein-based tablets can be designed using small-molecule pharmaceutical principles, supporting the development of oral protein formulations (contribution 7).

Clémence et al. reported that oral somatostatin receptor ligands (SRLs) like octreotide capsules (OOCs) and paltusotine show promise in acromegaly management, maintaining biochemical control in patients previously stabilized on injectable SRLs, as evidenced by recent trials (MPOWERED, PATHFNDR-1). While OOCs demonstrate long-term efficacy, gaps in the research remain—including direct drug comparisons and trials in SRL-naïve patients—highlighting the need for further research to optimize oral SRLs as a viable alternative to injections (contribution 8).

Xinxin et al.’s review article highlighted the potential for nano-formulations (liposomes, self-emulsifying systems, polymer particles) to enhance the oral delivery of antidiabetic peptides, offering promising solutions to improve gastrointestinal absorption and therapeutic efficacy (contribution 9).

Wanneng et al. introduced the gut microbiota, which plays a critical role in human health, and its regulation presents both therapeutic potential and challenges for treating disease. Additionally, they introduced the associated nanomaterials, with their protective, targeted, and biocompatible properties, demonstrated their significant promise in advancing the modulation of the gut microbiota, as highlighted by recent research and future development prospects (contribution 10).

Dan et al.’s review was focused on oxytocin, known for enhancing social cognition and reducing anxiety, and which is being explored as a therapy for social dysfunction disorders. While intranasal administration yielded a higher oxytocin bioavailability (11.1%) compared to oromucosal routes (4.4%), both methods showed similar peak concentration times (approximately 30 min) and comparable behavioral effects, highlighting the therapeutic potential of oromucosal delivery for oxytocin and other peptides in clinical and broader pharmaceutical applications (contribution 11).

Sílvio et al. discussed how inflammatory bowel disease (IBD) remains challenging to treat, but highlighted that probiotics show promise in managing symptoms, particularly for ulcerative colitis, though their stability and delivery need improvement. While the micro- and nanoencapsulation of probiotics and drugs enhances targeted colon delivery, overcoming gastrointestinal tract barriers and offering a novel therapeutic strategy for IBD, further clinical validation is needed for their long-term safety and efficacy (contribution 12).

Lastly, we would like to take this opportunity to thank all of the scientific researchers for their contributions to this Special Issue. While the oral delivery of protein/peptide drugs remains a formidable challenge, interdisciplinary advances in formulation science, nanotechnology, and molecular biology are driving progress in this field. Success in this area could revolutionize treatment by offering patients a more convenient and non-invasive alternative to injections, improving adherence and quality of life. Moreover, success hinges on balancing bioavailability, safety, and scalable manufacturing—a frontier requiring collaboration across pharmaceutics, biochemistry, and regulatory sciences. Continued research and collaboration are essential to bring these advanced therapies to the market.
